# Establishing rapid analysis of Pu isotopes in seawater to study the impact of Fukushima nuclear accident in the Northwest Pacific

**DOI:** 10.1038/s41598-018-20151-4

**Published:** 2018-01-30

**Authors:** Wu Men, Jian Zheng, Hai Wang, Youyi Ni, Tatsuo Aono, Sherrod L. Maxwell, Keiko Tagami, Shigeo Uchida, Masatoshi Yamada

**Affiliations:** 1 Biospheric Assessment for Waste Disposal Team, National Institute of Radiological Sciences, National Institutes for Quantum and Radiological Science and Technology, 491 Anagawa, Inage, Chiba 263-8555 Japan; 2 Laboratory of Marine Isotopic Technology and Environmental Risk Assessment, Third Institute of Oceanography, State Oceanic Administration, Xiamen, 361005 China; 3 Fukushima Project Headquarters, National Institute of Radiological Sciences, Anagawa 4-9-1, Inage, Chiba 263-8555 Japan; 4 School of Nuclear Science and Technology, University of South China, Hengyang, 421001 China; 5 Savannah River National Laboratory, Building 735-B, Aiken, SC 29808 USA; 6 Department of Radiation Chemistry, Institute of Radiation Emergency Medicine, Hirosaki University, 66-1 Hon-cho, Hirosaki, Aomori 036-8564 Japan

## Abstract

In order to assess the impact of the Fukushima derived Pu isotopes on seawater, a new analytical method to rapidly determine Pu isotopes in seawater by SF-ICP-MS including Fe(OH)_2_ primary co-precipitation, CaF_2_/LaF_3_ secondary co-precipitation and TEVA+UTEVA+DGA extraction chromatographic separation was established. High concentration efficiency (~100%) and high U decontamination factor (~10^7^) were achieved. The plutonium chemical recoveries were 74–88% with the mean of 83 ± 5%. The precisions for both ^240^Pu/^239^Pu atom ratios and ^239+240^Pu activity concentrations were less than 5% when 15 L of seawater samples with the typical ^239+240^Pu activity of the Northwest Pacific were measured. It just needs 12 hours to determine plutonium using this new method. The limit of detection (LOD) for ^239^Pu and ^240^Pu were both 0.08 fg/mL, corresponding to 0.01 mBq/m^3^ for ^239^Pu and 0.05 mBq/m^3^ for ^240^Pu when a 15 L volume of seawater was measured. This method was applied to determine the seawater samples collected 446–1316 km off the FDNPP accident site in the Northwest Pacific in July of 2013. The obtained ^239+240^Pu activity concentrations of 1.21–2.19 mBq/m^3^ and the ^240^Pu/^239^Pu atom ratios of 0.198–0.322 suggested that there was no significant Pu contamination from the accident to the Northwest Pacific.

## Introduction

Plutonium (Pu) is present in the marine environment mainly as a result of human activities related to atmospheric nuclear weapon tests, nuclear fuel reprocessing and nuclear accidents. From the viewpoint of radio-toxicity and long-term radiation effects to humans, Pu is by far one of the most important transuranic elements that have been released into the environment from nuclear plant accidents^1^. The element has twenty isotopes with mass ranging from 228 to 247. Among them, ^240^Pu and ^239^Pu are the most important due to their long half-lives(6537 y and 24100 y, respectively) and high abundance. As the result, ^239+240^Pu activity concentration can serve as an excellent tracer for studying sedimentary processes^2,3^, scavenging processes^4^, ocean current pathway^5^, particle transportation^6^ and other oceanic processes^7^. Moreover, ^240^Pu/^239^Pu atom ratio is of great interest because it has been well characterized for various sources, applied widely as a fingerprint to identify radioactive contamination sources in marine and terrestrial environments^6,7^. For instance, ^240^Pu/^239^Pu atom ratio of weapons-grade Pu ranges from 0.02 to 0.05, while nuclear reactor-grade ^240^Pu/^239^Pu ranges from 0.2 to 1.0 depending on irradiation conditions of the fuel^8^. The worldwide integrated global fallout ^240^Pu/^239^Pu is characterized by ratios of 0.17–0.19 with the average of 0.176 ± 0.014^9^. While the Pacific Proving Grounds (PPG) derived ^240^Pu/^239^Pu atom ratio is greater than 0.30^10^, which is close to that from the Fukushima Dai-ichi Nuclear Power Plant (FDNNP) with the value of 0.303–0.333^11,12^.

Approximately 15 PBq of ^239+240^Pu were released into the environment from atmospheric weapon tests conducted during the period of the 1950 s and 1960 s, and a few GBq of Pu were released to the marine environment from fuel reprocessing plants up until the present time^1^. It is reported that the total amount of ^239+240^Pu released into the ocean was ~12 PBq by the year 2000^13,14^. For the Pacific Ocean, it was estimated that total ^239+240^Pu deposited from the atmospheric nuclear weapon testing is around 8.6 PBq, which included 5.1 PBq of ^239+240^Pu in local and regional fallout^14^. The total seawater volume is ~1.37 × 10^18^ m^3^ ^15^, therefore, the resulting concentration of ^239+240^Pu in seawater is extremely low (~mBq/m^3^ or ~fg/L level). Moreover, the decreasing trend of ^239+240^Pu activity concentration with an apparent half-residence time of 5–21 y in surface seawater of the world’s oceans since 1970 is still unchanged^16–19^. The ^239+240^Pu activity concentrations in the surface seawater of the North Pacific decreased more than ten-fold from the early 1970 s (8.1–35 mBq/m^3^) to the year 2000 (0.3–2.7 mBq/m^3^)^16^. This makes measuring the current activity concentration of Pu in seawater very challenging. If the seawater samples were measured by a conventional radiometric analysis technique such as alpha-spectrometry, which counts the “dying” Pu atoms, a large volume of seawater (~200 L) and long analysis times (1–2 weeks) are typically needed. Meanwhile, alpha-spectrometry has the disadvantage of lacking the capability to provide the ^240^Pu/^239^Pu atom ratio, since these isotopes have similar alpha particle energies. By contrast, mass spectrometry, an atom-counting technique, has several advantages. It can be utilized with a small sample volume (several to tens of liters), simple sample preparation, short detection time, high sensitivity and precision as well as availability of accurate isotope ratio information, which is ideal for the determination of Pu isotopes in seawater.

Various analytical methods based on mass spectrometry have been developed including thermal ionization mass spectrometer (TIMS), accelerator mass spectrometer (AMS) and inductively coupled plasma mass spectrometry (ICP-MS)^1,20,21^. Among the mass spectrometric methods, ICP-MS is one of the most frequently employed for the last decade^22^. In recent years, the evolution of a new SF-ICP-MS with Jet Interface has developed for ultra-trace determination of Pu from femtogram (10^−15^ g) level to attogram (10^−18^ g) level. This allows comparable or even better sensitivity and detection limit than AMS^23^, which is ideal for the rapid determination of Pu isotopes in small volume seawater samples.

Even when the highly sensitive SF-ICP-MS is applied to determine Pu isotopes in seawater samples, the plutonium in the small volume seawater samples needs to be pre-concentrated. In addition, due to the serious mass interferences caused by the peak tailing effect of ^238^U^+^ (3.3 ng/ml in seawater, 9–10 orders of magnitude higher than that of Pu) and formation of uranium hydrides (^238^UH^+^ and ^238^UH_2_^+^), the ^238^U concentration in the final sample solution generally needs to be reduced to less than 5 pg/ml. To accomplish this removal of ^238^U, the U decontamination factor greater than ~10^7^ is needed^8,24^. Therefore, the analytical procedure such as co-precipitation, anion-exchange chromatography, extraction chromatography and the combination of these methods have been taken in recent years for the determination of Pu in seawater^24,25^.

The FDNPP accident released a great amount of artificial radionuclides into the Pacific Ocean^26^. It is reported that 1.0–2.4 GBq of ^239+240^Pu was released into the terrestrial environment^11,12^. As considerable amounts of highly contaminated effluents originating from the inner structure of the reactor have been emitted into the ocean^26,27^, addition Pu isotopes derived by FDNPP accident might have entered into the Pacific Ocean. It is necessary and important to continue monitoring the Pu isotopes in seawater for long-term radiological assessment. However, many current analytical techniques for Pu isotopes in seawater are inadequate, not only due to the difficulties in sampling large volumes of seawater, but also due to the complexities of the chemical treatments required. In addition, many current analytical methods cause quite lot of waste acids enhancing great burdens for laboratory management. Therefore, a rapid analytical method of small-volume seawater samples is highly desired not only for emergency response and assessment, but also for improving sample throughput and reducing environmental hazards in routine analyses. In this work, a new analytical method to rapidly determine Pu isotopes in seawater by SF-ICP-MS including a Fe(OH)_2_ primary co-precipitation, CaF_2_/LaF_3_ secondary co-precipitation and TEVA+UTEVA+DGA extraction chromatographic separation was established. And the activity concentrations of ^239+240^Pu isotopes as well as ^240^Pu/^239^Pu atom ratios in the surface water of Northwest Pacific were determined based on this new method to assess the impacts of the Fukushima derived Pu isotopes on the marine environment.

## Materials and Methods

### Reagents and seawater samples

A Millipore Milli-Q-Plus water purification system was used for the preparation of high-purity water (>18.2 MΩ/cm). HNO_3_ (60–61%), HCl (35.0–37.0%), HF (49.5–50.5%), NH_3_H_2_O (25.0–27.9%), TiCl_3_ (20%), FeCl_3_·6H_2_O, C_6_H_8_O_6_ (VC, Ascorbic acid), NaNO_2_, and H_3_BO_3_ are of analytical grade and obtained from Kanto Chemical Co., INC, Japan. Ca(NO_3_)_2_·4H_2_O and La(NO_3_)_2_·6H_2_O are also of analytical grade purchased from by Wako Pure Chemical Industries, Ltd, Japan. Analytical-grade Iron (II) sulfamate (38–42%) aqueous solution are obtained from Strem Chemicals, USA. Ultrapure-grade HNO_3_ (68%) is used for sample preparation prior to ICP-MS measurement, which is purchased from Tama Chemicals, Japan. Three kinds of extraction resins TEVA, UTEVA and DGA (Eichrom Technologies, LLC) used in this study were 2 mL cartridges with grain sizes of 50–100 μm. ^242^Pu (CRM 130, plutonium spike assay and isotopic standard, New Brunswick Laboratory, USA), as a yield tracer, was used to spike the seawater samples. IAEA-443 certified seawater reference material was obtained from the International Atomic Energy Agency (IAEA, Vienna, Austria).

Surface seawater samples (~17 L for each sample collected from 0–1 m from the surface) were collected from 446–1316 km off the FDNNP site in the Northwest Pacific during the cruises of MR 13–04 in July 2013, which were used to assess the impact of Fukushima derived Pu isotopes on seawater. After sampling, seawater samples were acidified to pH ~2 with concentrated HCl and then filtered into a HDPE bucket (20 L) (Teraoka Company, Japan). Then the seawater samples were transferred to the land-based laboratory.

### Seawater sample preparation

**Figure 1:**
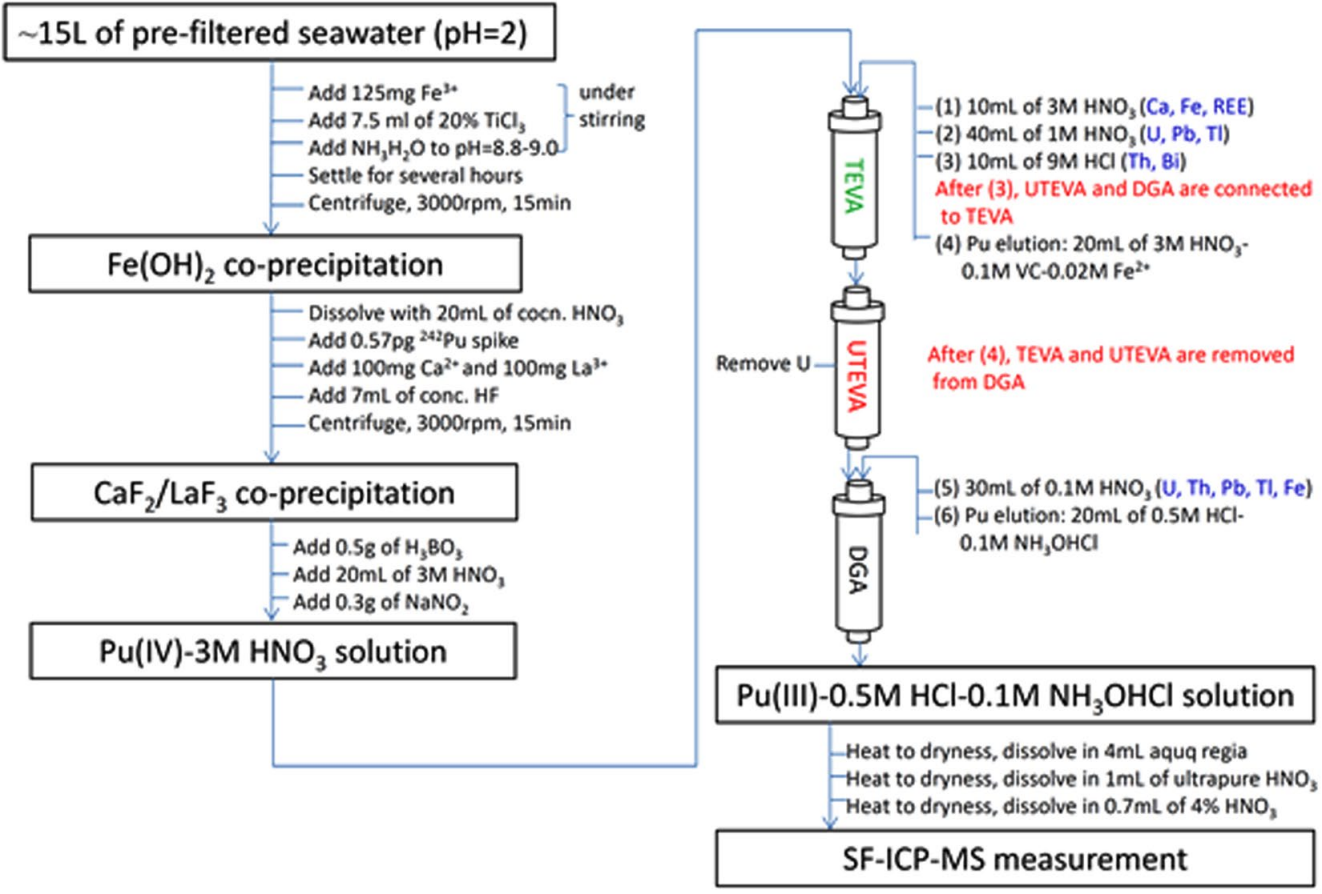
Flow chart of the analytical procedure for the determination of Pu isotopes in seawater by extraction chromatography and SF-ICP-MS

Prior to the analysis by SF-ICP-MS, seawater samples need a series of separation and purification to remove the matrix and interfering elements. The overall procedure is shown in Fig. 1.

### **Fe(OH)**_**2**_**co-precipitation**

Transfer ca. 15 L of pre-filtered seawater into a plastic container. TiCl_3_ (0.5 mL of 20% per liter seawater) and 125 mg Fe^3+^ (5 mL of 25 mg/mL) were added to the sample under stirring in sequence. Fe was used as a carrier, and TiCl_3_ was used to reduce Pu (V, VI) to Pu (III) for complete co-precipitation of Pu with iron hydroxide. The concentrated NH_3_H_2_O was added to adjust pH = 8.8–9.0 for Fe(OH)_2_ co-precipitation under stirring. The formed Fe(OH)_2_ co-precipitates were stirred at least 30 mins by stirrers and then allowed to settle for about 4 hours. The sample supernatant were carefully siphoned and pumped away avoiding loss of Fe(OH)_2_ co-precipitates. The obtained Fe precipitate slurry (ca. 400 mL) was collected in two 250 mL centrifuge tubes and the small volumes (ca. 50 mL) of pure water were used to rinse the sample bucket, which was also combined into the 250 mL centrifuge tubes. The slurry was centrifuged under 3000 rpm for 15 min. After pouring out the supernatant, 20 mL of concentrated HNO_3_ and 0.57 pg of ^242^Pu spike were added into the first centrifuge tube to dissolve the precipitate, and then the obtained solution was transferred into the second centrifuge tube to dissolve the rest of precipitate. Rinse the first centrifuge tube with 5 mL of deionized water and pour the rinsing water into the second centrifuge tube. The final solution was ca. 3.4 M HNO_3_ solution with the volume of ca. 75 mL.

### **CaF**_**2**_**/LaF**_**3**_**co-precipitation**

In order to form CaF_2_/LaF_3_ co-precipitation, 100 mg Ca^2+^, 100 mg La^3+^ and 7 mL of concentrated HF were added into the second centrifuge tube, followed by vigorous shaking and 15-minute settling. After centrifugation under 3000 rpm for 15 min, the supernatant was discarded and the precipitate was dissolved by 20 mL of 3 M HNO_3_ with the addition of 0.5 g of H_3_BO_3_. The solution was transferred into the 50 mL centrifuge tube and Pu (III) was adjusted to Pu (IV) by the addition of 0.3 g of NaNO_2_ and heated at 40 °C for 0.5 h in a temperature–controllable heating apparatus (DigiPREP Jr, SCP SCIENCE, Canada).

### Separation and purification

As shown in Fig. 1, the sample solution was loaded onto a TEVA resin cartridge (at a flow rate of 1 drop per second) which had been preconditioned with 10 mL of 3 M HNO_3_. An additional 10 mL of 3 M HNO_3_ was used to rinse the 50 ml centrifuge tube and leached through the TEVA resin cartridge to remove Ca, Fe, and rare earth elements (REEs), followed by 40 mL of 1 M HNO_3_ to remove U, Pb, and Tl, and 10 mL of 9 M HCl to remove Th, and Bi (at a flow rate of 2 drops per second). Before the elution of Pu, an UTEVA+ DGA resin cartridge (also preconditioned by 10 mL of 3 M HNO_3_) was connected to the bottom of the TEVA resin cartridge. Then 20 mL of 3 M HNO_3_ −0.1 M ascorbic acid −0.02 M Fe^2+^ (iron(II) sulfamate) was employed to reduce Pu (IV) to Pu (III) and elute Pu (III) from TEVA resin (1 drop per second) to the DGA resin. Then, the TEVA and UTEVA resin cartridges were removed. The DGA resin cartridge was rinsed by 30 mL of 0.1 M HNO_3_ to remove U, Tl, Pb, and Fe (2 drops per second). Next, the plutonium on the DGA resin was eluted into a 50 mL PTFE beaker by 20 mL of 0.5 M HCl −0.1 M NH_2_OH·HCl (1 drop per second). The eluted sample solution was evaporated to dryness on a hot plate with the temperature of 250 °C and dissolved using 4 mL of aqua regia. After heating the dissolved sample solution to dryness on a hot plate with the temperature of 200  °C, 1 mL of ultrapure HNO_3_ was added and heated to near dryness at 200 °C. Finally, the sample was dissolved in 0.7 mL of 4% HNO_3_ and ready for SF-ICP-MS measurement.

### SF-ICP-MS measurements

The measurement of Pu isotopes was carried out using a SF-ICP-MS (Element XR, Thermo Fisher Scientific Inc., Germany). An APEX-Q high efficiency sample introduction system (Elemental Scientific Inc, USA) combined with a membrane desolvation unit (ACM) and equipped with a conical concentric nebulizer (~0.15 mL/min) was used for sample introduction. A ^238^U standard solution (0.02 ng/mL) was used to adjust optimum performance daily before sample detection. The low resolution mode (m/Δm = 400) was used in order to utilize the maximum instrument sensitivity. The instrument and data acquisition settings of APEX-Q/SF-ICP-MS were detailed elsewhere^23^.

## Results and Discussion

### Pu valence adjustment and co-precipitation

The dissolved Pu isotopes in seawater are present primarily as Pu (V) or Pu (VI), which do not undergo co-precipitation as favorably as Pu (III) and Pu (IV). Therefore converting Pu to a reduced oxidation state is necessary to enhance the efficiency of co-precipitation^1^. Among the specific reducing agents NH_3_OHCl, Na_2_SO_3_, K_2_S_2_O_5_ and TiCl_3_^28–30^, TiCl_3_ was demonstrated having not only the ability of enhancing chemical recovery but also the advantage of being removed in the subsequent fluoride co-precipitation step^25^. As for the primary co-precipitation of Pu isotopes in seawater, MnO_2_ and Fe(OH)_3_ methods are frequently employed^25,28,29^. Compared to Fe(OH)_3_, which can be easily removed in the subsequent fluoride removal step, the removal of Mn from the final precipitates is somewhat more troublesome^25,31^. In addition, smaller amounts of reagents are required when applying the Fe(OH)_3_ co-precipitation compared with the MnO_2_ method. Actually, during the Fe(OH)_3_-primary co-precipitation in our method, TiCl_3_ reduces Fe^3+^ to Fe^2+^. Hence, the final co-precipitate here is Fe(OH)_2_.

For the need of further concentration, secondary co-precipitation is necessary. For the small volume of samples, rare-earth fluoride methods are extremely suitable for the co-precipitation of Pu and Pu separated from a supernatant solution containing U, Pt, Fe, Cr, etc.^32,33^. It was reported that LaF_3_ or NdF_3_ can selectively co-precipitate Pu (III, IV) effectively^21,25,34^. Another advantage of CaF_2_/LaF_3_ co-precipitation is that 60% of U can be removed by this procedure^35^. Besides, the fluoride co-precipitation can also remove the large amount of iron and titanium used in the primary co-precipitation step^25^. Therefore, in this study, CaF_2_/LaF_3_ was selected to for the secondary co-precipitation.

### **Pu concentration efficiency of Fe(OH)**_**2**_**co-precipitation**

^242^Pu is one of nuclear fuel nuclides. There are very strict management rules for the use of nuclear fuel nuclides in Japan^36^. It is controlled in some specified area and not allowed to take to the laboratory onboard, which limits its use for tracing Pu in-site pre-concentration on board and enhance the burden of sample transportation. In order to solve this problem, we attempted to study the feasibility of adding ^242^Pu spike after the primary Fe(OH)_2_ co-precipitation rather than at the beginning of experiment into the seawater samples directly. To this end, a set of experiments were designed to estimate the Pu concentration efficiency (CE) (ratio of Pu amount in the Fe(OH)_2_ co-precipitation to that in the original seawater sample) of Fe(OH)_2_ co-precipitation and its stability. First, we used primary co-precipitation procedure in Fig. 1 to remove the original ^239+240^Pu (unknown amount) from seawater and keeping the supernatant to get Pu-free seawater sample (15–16.6 L). Second, we added the 2.5–20 mL of IAEA-443 standard seawater into the Pu-free seawater sample to obtain seawater samples with very accurate known ^239+240^Pu amounts, which can make sure that we get the accurate recovery more scientifically. After the primary co-precipitation with Fe(OH)_2_, 0.57 pg of ^242^Pu spike were added into the solution before the CaF_2_/LaF_3_ co-precipitation. So the final results of ^239+240^Pu we calculated represented the ^239+240^Pu activity concentration of the IAEA-443 standard added seawater without an estimation of Pu recovery during Fe(OH)_2_ co-precipitation. If the value was divided by the certified ^239+240^Pu activity concentration of added IAEA-443 reference seawater, the Pu CE of Fe(OH)_2_ co-precipitation can be estimated. As shown in Table 1, Pu CE of Fe(OH)_2_ co-precipitation ranged from 98.8% to 103% with an average of 101 ± 2% suggesting that Fe(OH)_2_ co-precipitation can quantitatively recover Pu isotopes in seawater. In addition, all of the CEs were consistent in the range of 2σ standard deviation (Table 1), which demonstrated its reliable stability for concentrating Pu isotopes from seawater. In other words, the loss of Pu during Fe(OH)_2_ co-precipitation is negligible. So our results confirmed that this method is practical.

**Table 1:** Pu concentration efficiencies of Fe(OH)_2_ co-precipitation.

Sample	Seawater volume (L)	Added IAEA443 (mL)	Measured ^240^Pu/^239^Pu	Certified ^240^Pu/^239^Pu	Measured ^239+240^Pu (mBq/m^3^)	Certified ^239+240^Pu (mBq/m^3^)	Concentration efficiency of Fe(OH)_2_ (%)
1	15	17.5	0.233 ± 0.018	0.229 ± 0.006	19.0 ± 1.2	18.6 ± 1.4	103
2	16.6	20	0.225 ± 0.009	0.229 ± 0.006	17.0 ± 0.8	17.2 ± 0.5	99.1
3	15	15	0.230 ± 0.016	0.229 ± 0.006	14.6 ± 1.2	14.6 ± 0.4	100
4	15	12.5	0.238 ± 0.028	0.229 ± 0.006	13.6 ± 1.4	13.3 ± 1.0	102
5	15	10	0.239 ± 0.026	0.229 ± 0.006	10.8 ± 0.7	10.6 ± 0.8	102
6	15	7.5	0.238 ± 0.033	0.229 ± 0.006	8.19 ± 0.86	7.95 ± 0.61	103
7	15	5	0.241 ± 0.040	0.229 ± 0.006	5.24 ± 0.60	5.30 ± 0.41	98.8
8	15	2.5	0.236 ± 0.040	0.229 ± 0.006	2.70 ± 0.27	2.65 ± 0.20	102

As a matter of fact, in addition to the advantage of in–site processing seawater samples, there is another advantage of our method. It can greatly reduce the radioactive wastes that contains ^242^Pu and the resulting burden of waste disposal for laboratories with restrictions on the use of nuclear fuel nuclides.

### Pu chemical recovery and decontamination factor of U

Chemical separations of Pu are commonly carried out using anion exchange resins or extraction chromatography. Anion-exchange resins such as AG 1 × 8, AG MP-1M and Dowex 1 × 8 have attracted much attention for their low cost and wide applicability as well as strong tolerance capacity for matrix elements^1,22^. On the other hand, extraction resins such as TEVA, UTEVA, TRU and DGA have the advantages of short sample processing time, high recovery and less amount of acidic wastes. It is worth noting that, the prerequisite for using extraction resins for Pu separation is that large amounts of matrix elements must be removed almost completely through suitable pretreatment^22^. TEVA resin has a high capacity factor for Pu (3 × 10^4^) and is more effective for samples containing high concentrations of Fe, Mn, Ce, and interfering elements, such as U, Pb, Hg, which become the first choice for Pu separation^8,35^. UTEVA resin has much higher selectivity for U^8^. DGA resin can retain both trivalent and tetravalent actinides and further remove U, Tl, Pb, and Fe^34,35^. It is reported that the decontamination factor (DF) of U up to ~3 × 10^6^ can be achieved for the urine sample when TEVA+UTEVA+DGA resin combination was used^25^. It met the requirements of U DF for Pu determination in seawater. Therefore, based on the reason abovementioned, we choose the TEVA+UTEVA+DGA resins combination for Pu separation in seawater in this study.

Pu chemical recovery is one of the crucial indicators to assess the effectiveness of a Pu determination method. A high recovery is essential for the determination of Pu in small volume seawater sample, because the amount of Pu in seawater samples is not much higher than the detection limit of the SF-ICP-MS. It was found that the signal intensity of ^240^Pu atom was just around 50 ~ 80 cps when we measured the surface seawater (15–20 L) of Northwest Pacific, which suggested that the small fluctuation of recovery might result in a large influence on the accuracy and precision of the result. Lower recovery could result in a failure to analyze the ^239+240^Pu activity concentration and ^240^Pu/^239^Pu atom ratio. In addition to Pu chemical recovery, the DF of U is another crucial indicator to assess the Pu determination method. For the ultra-trace analysis of Pu isotopes, even though there is only a micro interference of uranium, it will hinder accuracy and precision of the Pu activity concentrations and their atom ratios.

To better understand the Pu chemical recovery and DF of U of our method, we repeated the Pu measurement (n = 8) for the same seawater sample collected from the Northwest Pacific. The seawater sample (120 L) were divided into 8 subsamples with the volume of 15 L each. The results are shown in Table 2. Pu chemical recovery of this method is 74–88% with a mean of 83 ± 5% (n = 8). U DF is 1.6 × 10^6^ ~ 2.4 × 10^7^ with a mean of (1.5 ± 0.9) × 10^7^. Table 3 lists the comparison of our method with the reported analytical methods of seawater including Pu chemical recovery, DF of U, limit of detection (LOD) and so on. As shown in Table 3, Pu chemical recovery of this method was comparable to that of the other Pu analysis methods of seawater, which suggested that the satisfactory chemical recovery was achieved in our new method.

**Table 2:** Results of repeated measurements (n = 8) for the same seawater sample.

Sample	Recovery (%)	DF of U	^240^Pu/^239^Pu	^239+240^Pu (mBq/m^3^)
A1	78 ± 7	2.4 × 10^7^	0.231 ± 0.027	1.83 ± 0.21
A2	87 ± 8	2.2 × 10^7^	0.254 ± 0.040	1.78 ± 0.28
A3	88 ± 7	1.6 × 10^6^	0.238 ± 0.041	1.71 ± 0.28
A4	87 ± 7	2.7 × 10^6^	0.234 ± 0.031	1.91 ± 0.25
A5	86 ± 8	6.7 × 10^6^	0.256 ± 0.034	1.94 ± 0.26
A6	87 ± 9	1.3 × 10^7^	0.263 ± 0.046	1.86 ± 0.33
A7	74 ± 7	7.1 × 10^6^	0.243 ± 0.035	1.85 ± 0.27
A8	80 ± 9	1.5 × 10^7^	0.239 ± 0.035	1.97 ± 0.29
Mean ± std	83 ± 5	1.5 × 10^7^ ± 0.9 × 10^7^	0.245 ± 0.023 (*k* = 2)	1.86 ± 0.17 (*k* = 2)

**Table 3:** Comparison of different analytical methods of Pu isotopes in seawater.

Separation	Volume	Recovery	DF for U	Measurement method	LOD (fg/mL)		^240^Pu/^239^Pu ratio		^239+240^Pu		Conditions to obtain accuracy and presicion	Ref.
	L	%			^239^Pu	^240^Pu	Accuracy, %	Precision, %	Accuracy, %	Precision, %		
AG1×8+UTEVA-TRU	18–105	87	1.2 × 10^4^	MC-ICP-MS	0.02	0.02	—	1.3–2.6	—	—	IAEA 384 and IAEA-367 sediment, ^240^Pu 10–25 fg/mL, n = 10	^8^
TEVA-UTEVA	10	74–77	5.3 × 10^4^	ICP-MS	—	—	—	—	—	1.2	Atlantic seawater spiked ^239^Pu, 1120 mBq/m^3^, 10 L, n = 3	^28^
Dowex 1×8+Dowex 1×8	20–60	58–82	3 × 10^7^–1 × 10^8^	SF-ICP-MS	0.11	0.08	3.3	4.5	2.7	1.9	IAEA 443 spiked seawater, 36.5 mBq/m^3^, 20 L,n = 2	^24^
TTA-benzene	4700–10800	96	1.7 × 10^7^	ICP-MS	0.34	0.43	6.8	—	—	—	IAEA 381, 13700 mBq/m^3^, V = 500 mL, n = 3	^49^
AG1×4	200	60	1.2 × 10^4^	ICP-MS	—	—	3.9	13.1	4.6	8.9	IAEA 381, 13700 mBq/m^3^, V = 500 mL, n = 4	^50^
Sr, TEVA	3–10	65	1.2–2.4 × 10^6^	SF-ICP-MS	0.64	0.19	9.1	3.1	4.2	2.3	IAEA 381, 13700 mBq/m^3^, V = 100 mL, n = 10	^51^
TEVA+UTEVA+AG MP−1M	200	50–60	1 × 10^6^–10^7^	ICP-MS	10	10	—	—	—	≤ 15	Seawater, 2–6 mBq/m^3^, V = 200 L, n = 4	^52^
AG1–8×+TEVA	6–80	~70	—	ICP-MS	5	—	4.5	—	7.3		IAEA 381, 13700 mBq/m^3^, V and n = not mentioned	^53^
TEVA+TEVA	5–20	~80	—	SF-ICP-MS	1	—	9.1	—	4.4	—	IAEA 381, 13700 mBq/m^3^, V and n = not mentioned	^53^
UTEVA-TRU	1–20	—	—	ICP-MS	—	—	3.6	4.0	5.6	5.4	IAEA 381, 13700 mBq/m^3^, n = 4	^20^
UTEVA-TRU	1–20	—	—	AMS	—	—	10.0	—	12.6	—	IAEA 381, 13700 mBq/m^3^, n = 1	^20^
AG1-8×+ TEVA	—	94–107	—	ICP-MS	—	—	9.0	1.4	9.1	11.9	IAEA 381 spiked seawater,1370–2283 mBq/m^3^, V = 3–5 L, n = 3	^54^
XDA-2+Dowex 1×2	200–500	—	—	α-spectrometer	—	—	—	—	—	—	Quantitative recovery, no data for accuracy and precision	^55^
TEVA+UTEVA+DGA	~15	74–88	1.6 × 10^6^–2.4 × 10^7^	SF-ICP-MS	0.08	0.08	3.0	2.2			IAEA 443 spiked seawater, 2.65–18.55 mBq/m^3^, V = 15–16.6 L, n = 8	This study
TEVA+UTEVA+DGA	~15	74–88	1.6 × 10^6^–2.4 × 10^7^	SF-ICP-MS	0.08	0.08	—	4.6	—	4.7	North Pacific seawater, 1.86 mBq/m^3^, V = 15 L, n = 8	This study

As shown in Table 2, U DFs of our method (1.6 × 10^6^ ~ 2.4 × 10^7^ with the mean of 1.5 × 10^7^ ± 0.9 × 10^7^) are better than that of the most previously reported values in Table 3. As mentioned in the introduction section, a U DF of ~10^7^ means that Pu was successfully separated from U. Understandably, the ^238^U signal intensities in the final Pu fraction for the seawater sample detected by SF-ICP-MS were about 100,000 cps, similar to that of the operational blank. As the ^238^UH^+^/^238^U^+^ ratio for our SF-ICP-MS system was less than 3 × 10^−5^, the contribution of ^238^UH^+^ to the ^239^Pu^+^ of interest was below 3 cps. Considering that the intensity of ^239^Pu in the Northwest Pacific seawater samples (15–20 L, ca. 1 mBq/m^3^ for ^239+240^Pu) exceeded 230 cps, our method made the U interference negligible for determination of Pu isotopes in seawater.

### **Accuracy**, **precision and reproducibility of the analytical method**

**Figure 2:**
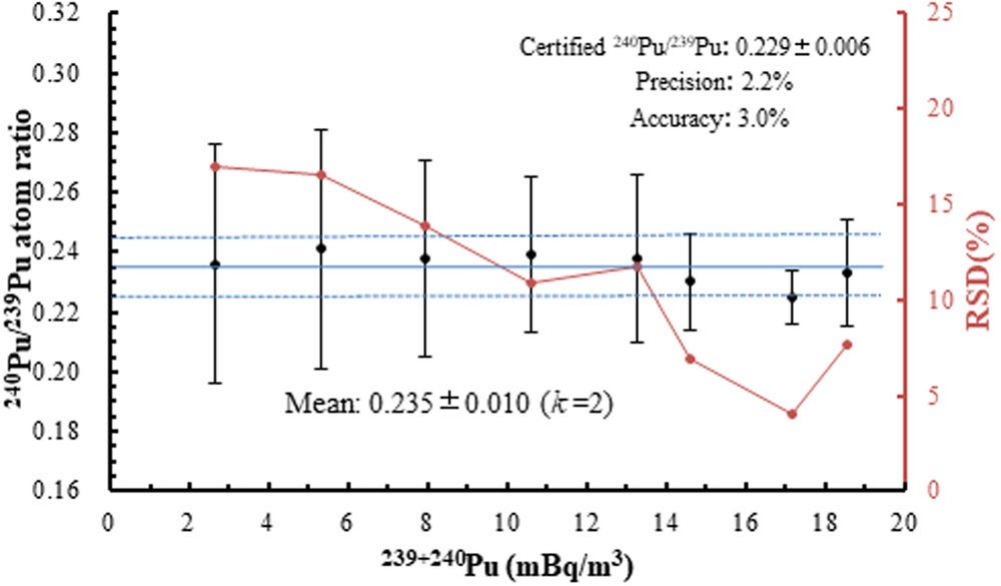
Accuracy and precision of ^240^Pu/^239^Pu atom ratio measurement obtained from serial IAEA-443 spiked samples. The error bars represent measuring error of each analysis. Horizontal solid and dashed lines represent the overall average ^240^Pu/^239^Pu atom ratios and expanded standard uncertainties (*k* = 2), respectively.

Accuracy and precision are the two essential indicators for the reliability of one analytical method. Figure 2 plots the data of samples in Table 1. It presented the measured results of IAEA 443-spiked seawater samples with different activity concentrations of ^239+240^Pu and the relationship between the precisions of single measurement of ^240^Pu/^239^Pu atom ratio with a serial ^239+240^Pu activity concentrations (2.65–18.55 mBq/m^3^). The mean of measured ^240^Pu/^239^Pu atom ratios (n = 8) was 0.235 ± 0.010 (*k = *2). Their precision (RSD %) and accuracy were 2.2% and 3.0%, respectively. The single measurement precision increased with increasing ^239+240^Pu activity concentrations ranging from 4.0% to 16.9%. However, the ^240^Pu/^239^Pu atom ratios remain within the 95% confidence level of the overall average, suggesting that this method is a suitable technique for accurate measurements of Pu isotopic ratio in seawater sample with ultra-trace concentrations of Pu. As for the precision of single measurement of seawater ^239+240^Pu activity concentrations (2.65–18.55 mBq/m^3^), they ranged from 4.9–11.4%.

**Figure 3:**
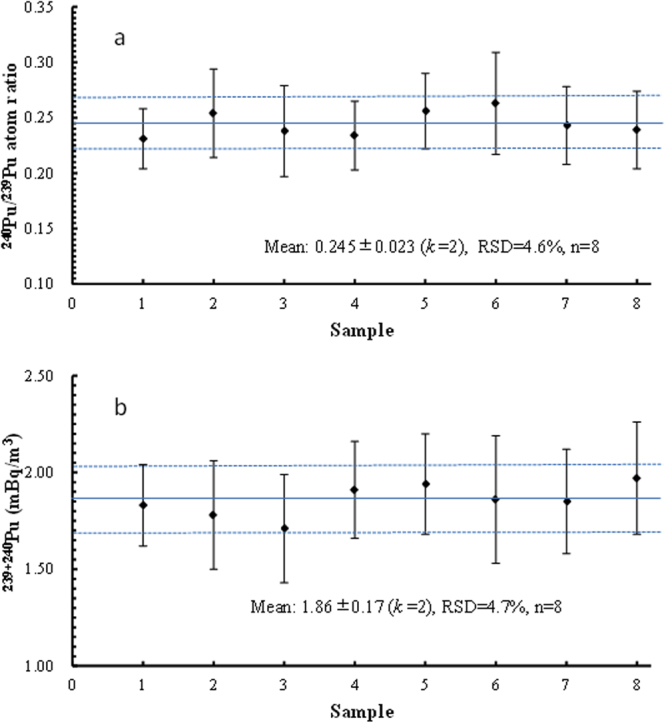
Precision of ^240^Pu/^239^Pu atom ratios (**a**) and ^239+240^Pu activity concentrations (**b**) measurement obtained from the Northwest Pacific seawater samples. The error bars represent measuring error of each analysis. Horizontal solid and dashed lines represent the overall average values and expanded standard uncertainties (*k* = 2), respectively.

We also evaluated the reproducibility of the entire analytical method by repeated measurements (n = 8) of ^239+240^Pu activity concentration and ^240^Pu/^239^Pu atom ratio in a seawater sample with a typical ^239+240^Pu activity concentration <2 mBq/m^3^ in Northwest Pacific. As shown in Fig. 3, for the 8 repeated measurements, the mean of ^239+240^Pu activity concentration was 1.86 ± 0.17 mBq/m^3^ (*k* = 2) and the mean of ^240^Pu/^239^Pu atom ratios was 0.245 ± 0.023 (*k* = 2). The precision of 8-time repeated measurement of ^240^Pu/^239^Pu atom ratios and ^239+240^Pu activity concentrations were 4.7% and 4.6%, respectively. The single measurement precision of the ^240^Pu/^239^Pu atom ratios and ^239+240^Pu activity concentrations ranged from 11.7–17.5% and 11.5–17.7%, respectively. All the single measurement results of ^239+240^Pu activity concentration and ^240^Pu/^239^Pu atom ratio varied within 2σ of the mean values. It suggested that this method can achieve a precision of <5% for both ^240^Pu/^239^Pu atom ratios and ^239+240^Pu activity concentrations when 15 L of seawater samples with the typical ^239+240^Pu activity concentration of the surface seawater in Northwest Pacific (~1.9 mBq/m^3^, corresponding to 9.8 fg/mL ^239^Pu and 2.2 fg/mL ^240^Pu in the final measured solution) are measured. In addition, as presented in Table 2, the Pu recovery ranged from 74% to 88%, with a mean of 83% ± 5%, indicating the analytical method is highly stable and reproducible. A detailed comparison with other published analytical methods for seawater Pu analysis is presented in Table 3. As shown in Table 3, the precision and accuracy for both ^239+240^Pu activity and ^240^Pu/^239^Pu atom ratio of our new method is comparable or better than most of the published methods.

### Limit of detection and validation of the method

High sensitivity and low detection limit are needed for the high performance analysis of Pu in small-volume seawater due to the low concentration of Pu. We used the APEX-Q sample introduction system to promote the sensitivity of SF-ICP-MS (Element XR). As described in previous work^23^, the sensitivity of the whole system can be improved up to 60Mcps/ppb, which can reduce greatly the volume of the samples.

The instrument detection limits of ^239^Pu and ^240^Pu were determined based on the estimation of 3 times the standard deviation of a 4% HNO_3_ blank solution. Similarly, the LODs of our method were calculated according to 3 times the standard deviation of the operation blanks. On the basis of analyzing 15 L of pure water with a Pu recovery of 83%, the LODs for ^239^Pu and ^240^Pu were both 0.08 fg/mL, corresponding to 0.01 mBq/m^3^ for ^239^Pu and 0.05 mBq/m^3^ for ^240^Pu when a 15 L of seawater was measured. As shown in Table 3, the LODs of this study is much lower than most of the reported LODs and comparable with Bu *et al*.^24^ but higher than that in the work reported by Lindahl *et al*.^8^. It should be noted that Lindahl *et al*.^8^ used 1% HNO_3_ solution to estimate the LODs. Their LODs were merely the MC-ICP-MS instrumental LODs, not the real LODs of entire analytical method. Therefore, the LODs of this study are among the lowest in the reported LODs.

The seawater reference material IAEA-443 was employed to validate this new method. A serial of IAEA-443 spiked seawater samples (2.5–20 mL of IAEA-443 in 15–16.6 L “Pu pre-removed” seawater) were used to illustrate the validation of our method. The results of ^240^Pu/^239^Pu atom ratios ranged from 0.225 ± 0.009 to 0.241 ± 0.040, which agreed well with the certificate values of 0.229 ± 0.006 within the range of error (Table 1). As for the ^239+240^Pu activity concentrations, each of them agreed well with the corresponding certificate value within the range of error (Table 1).

### Sample throughput

The complete analytical method takes about 12 h: Fe(OH)_2_ co-precipitation, 5 h; CaF_2_/LaF_3_ co-precipitation, 1 h; Pu separation on extraction resin, 2 h; and sample preparation for SF-ICP-MS measurements, 4 h. Compared to analytical methods employed conventional ion-exchange chromatographic separation, which usually takes about 3–5 days for Pu separation, the new method in this study significantly shortens the analytical time. If the vacuum box with 24 positions were used, 24 samples could be separated and purified simultaneously. It would be an extremely high sample throughput. In addition, this method produces less amount of hazardous acidic wastes and requires less evaporation of acids, greatly reduces the burden of radioactive laboratory management.

### Assessment on the Impact of Fukushima-derived Pu isotopes on seawater

**Figure 4:**
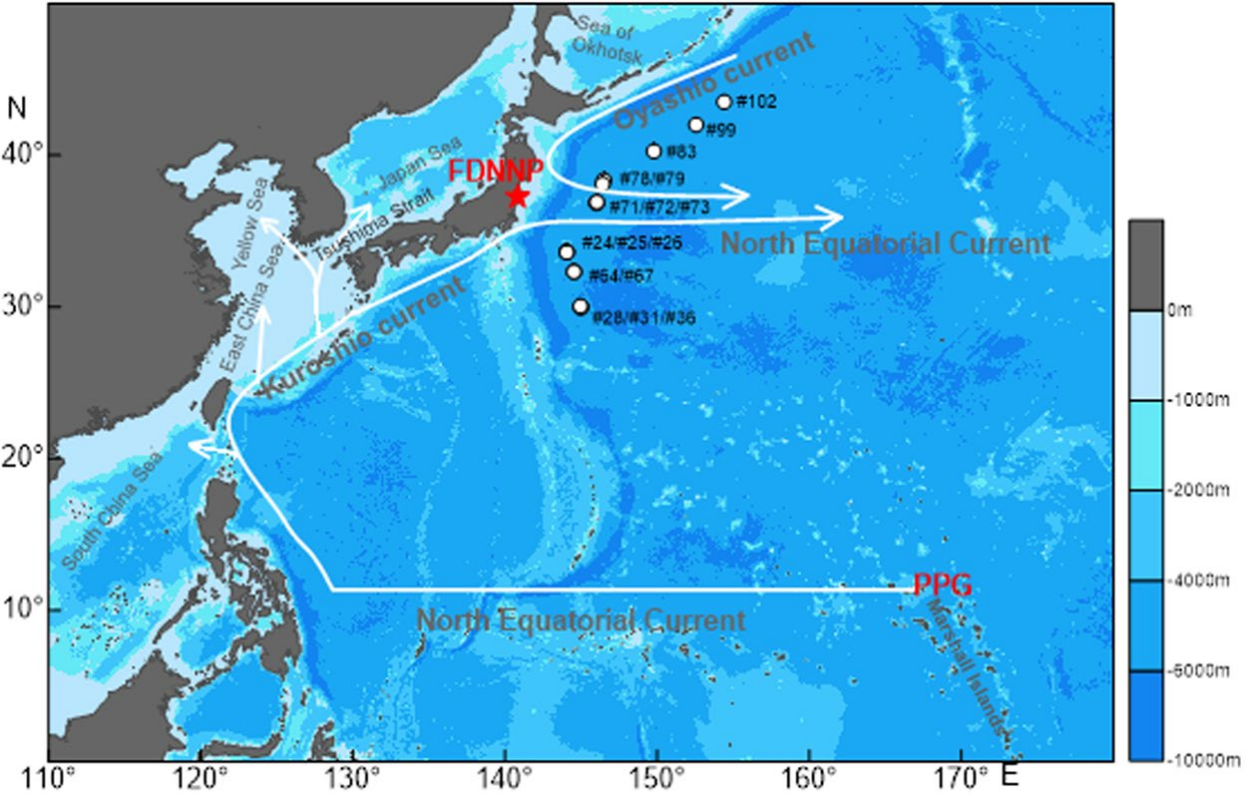
Surface seawater sampling stations in the Northwest Pacific in July, 2013. This figure was drawn using Surfer version 12.5.905 (http://www.goldensoftware.com).

**Figure 5:**
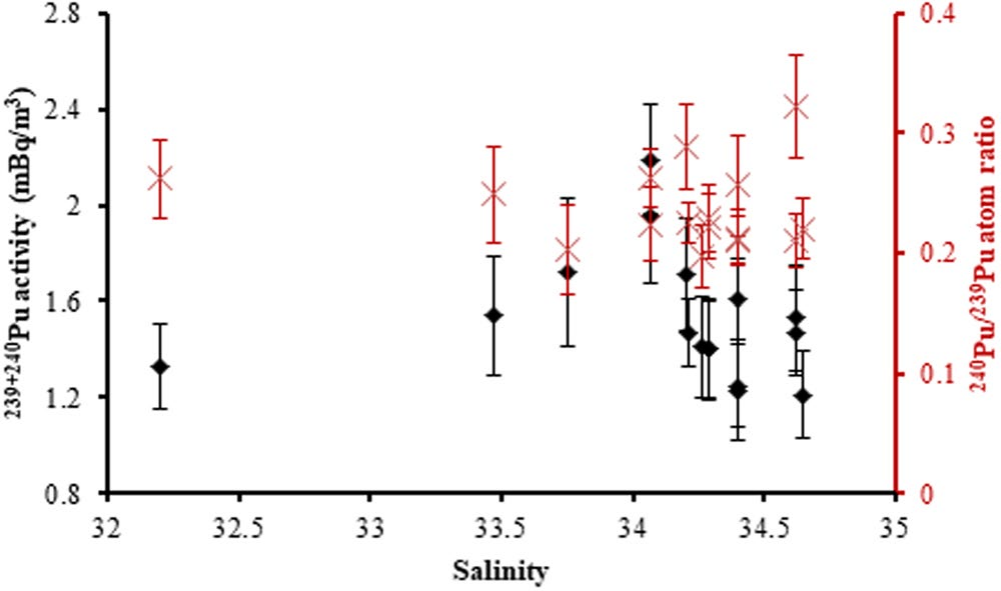
Relationships of ^239+240^Pu activity concentrations and ^240^Pu/^239^Pu atom ratios with salinities.

The developed method was applied to determine the ^239+240^Pu activity concentrations and ^240^Pu/^239^Pu atom ratios in 16 surface seawater samples obtained 446–1316 km off the FDNPP site in the Northwest Pacific in July 2013 (Fig. 4). The results are shown in Table 4 with other relevant oceanography information. The ^239+240^Pu activity concentrations ranged from 1.21 ± 0.18 to 2.19 ± 0.23 mBq/m^3^, showing that the value was within the range of pre-FDNPP accident period^16^. For ^240^Pu/^239^Pu atom ratios, values were from 0.198 ± 0.026 to 0.322 ± 0.042, respectively, also showing that they were within the range of the Northwest Pacific in pre-FDNPP accident period^37^. There were no obviously relationships of ^239+240^Pu activity concentrations and ^240^Pu/^239^Pu atom ratios with salinities (Fig. 5).

**Table 4:** ^239+240^Pu activity concentrations and ^240^Pu/^239^Pu atom ratios in surface seawater 446–1316 km off the FDNPP site.

Sample	Sampling date	Longitude (°E)	Latitude (°N)	Distance off FDNNP (km)	Temperature (°C)	Salinity	^239+240^Pu activity concentration (mBq/m^3^)	^240^Pu/^239^Pu atom ratio	Contribution of the PPG
#24	2013/7/13	144°02.7774′	33°38.2482′	501	25.824	34.399	1.61 ± 0.17	0.211 ± 0.019	22%
#25	2013/7/13	144°03.0808′	33°37.1140′	503	25.825	34.399	1.25 ± 0.17	0.213 ± 0.024	24%
#26	2013/7/13	144°03.5040′	33°35.5861′	506	25.830	34.398	1.23 ± 0.21	0.256 ± 0.042	50%
#28	2013/7/14	144°59.2774′	29°58.5172′	905	27.425	34.622	1.47 ± 0.18	0.210 ± 0.022	22%
#31	2013/7/14	144°59.1796′	29°58.4339′	905	27.375	34.623	1.53 ± 0.22	0.322 ± 0.042	84%
#36	2013/7/15	144°57.2963′	30°02.9320′	896	26.471	34.648	1.21 ± 0.18	0.220 ± 0.025	28%
#64	2013/7/17	144°31.8151′	32°18.8512′	651	26.682	34.289	1.40 ± 0.21	0.228 ± 0.028	34%
#67	2013/7/17	144°31.7644′	32°18.8257′	651	26.674	34.289	1.40 ± 0.20	0.222 ± 0.027	30%
#71	2013/7/18	146°00.7007′	36°50.3391′	446	25.221	34.208	1.47 ± 0.14	0.225 ± 0.017	32%
#72	2013/7/18	146°01.2901′	36°52.3403′	446	25.094	34.203	1.71 ± 0.23	0.289 ± 0.035	68%
#73	2013/7/18	146°01.9230′	36°54.5844′	446	25.091	34.266	1.41 ± 0.21	0.198 ± 0.026	13%
#78	2013/7/18	146°29.9774′	38°20.5436′	490	22.536	34.066	2.19 ± 0.23	0.262 ± 0.024	54%
#79	2013/7/18	146°24.9745′	38°05.6004′	479	22.520	34.065	1.95 ± 0.28	0.224 ± 0.031	31%
#83	2013/7/19	149°47.0870′	40°15.6761′	820	20.862	33.749	1.72 ± 0.31	0.203 ± 0.037	17%
#99	2013/7/20	152°33.5458′	42°00.3665′	1108	17.393	33.467	1.54 ± 0.25	0.249 ± 0.040	46%
#102	2013/7/20	154°24.0429′	43°30.6718′	1316	15.373	32.198	1.33 ± 0.18	0.262 ± 0.033	54%

**Figure 6:**
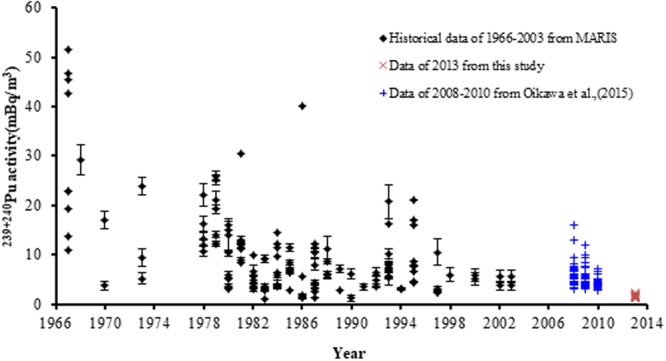
Comparison of ^239+240^Pu activities in this study with the historical ^239+240^Pu data in surface seawater of the Western North Pacific. (The data of area 15–40°N, 110–160°E in 1966–2003 are from IAEA-MARIS-Marine Information System^46^; the data of the commercial nuclear power station sites around the Japanese Island from 2008 to 2010 are from Oikawa *et al*.^18^.

After the FDNPP accident, ^239+240^Pu activity concentrations and ^240^Pu/^239^Pu atom ratios in seawater of Northwest Pacific were investigated to study if there was significant impact for Pu isotopes in seawater before and after the FDNPP accident. The seawater zone of <30 km, 30–200 km and >200 km off FDNNP were investigated respectively^19,24,38–40^. Their results suggested no significant Pu contamination from the accident in these areas. The ^240^Pu/^239^Pu atom ratios (0.198–0.322) were higher than the global fallout ratio of 0.176 ± 0.014^9^, but lower than that of the PPG nuclear weapon tests of 0.30–0.36^41–43^. Coincidentally, the FDNNP derived ^240^Pu/^239^Pu atom ratios had the similar range of 0.30–0.33 with that of PPG nuclear weapon tests^11^. Thus we could not conclude whether the FDNPP accident derived Pu isotopes had contributions to the 16 measured samples based on ^240^Pu/^239^Pu atom ratio in this study. Furthermore, ^241^Pu/^239^Pu atom ratios in the seawater should be measured because the FDNNP derived Pu isotopes had another characteristic ^241^Pu/^239^Pu atom ratios of 0.103–0.135^11^, which had much higher ^241^Pu/^239^Pu atom ratios compared to that of global fallout (0.00089) and PPG nuclear weapon tests (0.0017–0.0024)^38,39,44,45^. However, it is still difficult to measure the ^241^Pu in seawater at present. The relative technique remains to be further developed. However, the comparison of ^239+240^Pu concentrations before and after the FDNPP accident can be made to confirm whether it is above the range of that before FDNPP accident. Oikawa *et al*.^18^ reported the data of Pu isotopes in the surface seawater of the sites of commercial nuclear power stations around the Japanese Island from 2008 to 2010. In addition, the IAEA-MARiS-Maine Information System also records the variation of Pu isotopes in surface seawater of the Western North Pacific (15–40°N, 110–160°E) from 1966 to 2003^46^. All these data could be used as the baseline data before the FDNPP accident. The ^239+240^Pu activity concentrations of the zone of >400 km from the FDNPP in this study (1.21–2.19 mBq/m^3^) was typically in the range of the background data of the Northwest Pacific (Fig. 6)^18,19,24,46^, indicating negligible Pu contamination from the accident.

Wu *et al*.^47^ confirmed that even 60 years after the 1950 s, the PPG was still a dominant Pu source in the marginal seas of the Northwestern Pacific due to continuous transport of remobilized Pu from the Marshall Islands along the North Equatorial Current followed by the transport of the Kuroshio Current and its extension (Fig. 4). Now that abovementioned work has proved that there were negligible Pu isotopes released from the FDNPP accident. Therefore, that the ^240^Pu/^239^Pu atom ratios of this study were higher than the global fallout but lower than that of the PPG nuclear weapon tests suggested that the Pu isotopes mainly come from global fallout and PPG. We can estimate the contributions of global fallout and PPG close-in fallout Pu using the two end-member mixing model proposed by Krey *et al*.^9^:

1 $$\frac{{({}^{239+240}Pu)}_{P}}{{({}^{239+240}Pu)}_{G}}=\frac{({R}_{G}-{R}_{s})(1+3.674{R}_{P})}{({R}_{s}-{R}_{P})(1+3.674{R}_{G})}$$

2 $${({}^{239+240}Pu)}_{P}+{({}^{239+240}Pu)}_{G}=1$$

where R refers to ^240^Pu/^239^Pu ratio, subscripts P, G and S refer the PPG close-in fallout, and the global stratospheric fallout and the samples measured in this study, respectively. The value 3.674 is the ratio of the specific activity of ^240^Pu to ^239^Pu, by which the atom ratio is converted to the activity ratio. Krey *et al*.^9^ reported the ^240^Pu/^239^Pu atom ratio of global fallout to be 0.18 ± 0.02 between 30°N and 60°N. The close-in fallout in the PPG had a ^240^Pu/^239^Pu atom ratio of 0.363 ± 0.004^48^. The contributions of the PPG close-in fallout of this study were with the range of 13%-84% (Table 4). Buesseler^41^ suggested that Pu derived from the PPG would be preferentially removed from the water column, compared with the global stratospheric fallout Pu that is more soluble. Therefore the high contributions of PPG close-in fallout Pu (e.g. PPG contributions of 84% with the ^240^Pu/^239^Pu atom ratio of 0.322) in this study might suggest that the latest transport of the PPG-derived Pu to the corresponding area.

## Summary

In this study, a high-performance method for the determination of Pu isotopes in small volume seawater based on Fe(OH)_2_ pre-concentration, CaF_2_/LaF_3_ co-precipitation, TEVA+UTEVA+DGA extraction chromatographic separation and SF-ICP-MS measurement was reported. The Pu concentration efficiency of Fe(OH)_2_ co-precipitation was close to ~100%. Pu chemical recovery of the developed method ranged from 74–88% with the mean of 83 ± 5%. A high U decontamination factor of ~10^7^ was achieved, which made the U interference negligible for the determination of ultra-trace Pu isotopes. The LOD for ^239^Pu and ^240^Pu were both 0.08 fg/mL, corresponding to 0.01 mBq/m^3^ for ^239^Pu and 0.05 mBq/m^3^ for ^240^Pu when a 15 L volume of seawater was measured. The entire analytical method only took about 12 h. This method was applied to determine the seawater samples collected 446–1316 km off the FDNPP accident site in the Northwest Pacific in July of 2013. The ^239+240^Pu activity concentrations of 1.21–2.19 mBq/m^3^ and the ^240^Pu/^239^Pu atom ratios of 0.198–0.322 were obtained, which also suggested there was no significant Pu contamination from the accident to the Northwest Pacific.
